# Vector Field Embryogeny

**DOI:** 10.1371/journal.pone.0008177

**Published:** 2009-12-17

**Authors:** Till Steiner, Yaochu Jin, Bernhard Sendhoff

**Affiliations:** Honda Research Institute Europe GmbH, Offenbach am Main, Germany; Centre for Genomic Regulation (CRG), Universitat Pompeu Fabra, Spain

## Abstract

We present a novel approach toward evolving artificial embryogenies, which omits the graph representation of gene regulatory networks and directly shapes the dynamics of a system, i.e., its phase space. We show the feasibility of the approach by evolving cellular differentiation, a basic feature of both biological and artificial development. We demonstrate how a spatial hierarchy formulation can be integrated into the framework and investigate the evolution of a hierarchical system. Finally, we show how the framework allows the investigation of allometry, a biological phenomenon, and its role for evolution. We find that direct evolution of allometric change, i.e., the evolutionary adaptation of the speed of system states on transient trajectories in phase space, is advantageous for a cellular differentiation task.

## Introduction

Biological evolution has found ways to build organisms with astonishing complexity, where both, the number of elements and the robustness of interactions between them, often exceed those of engineered products. Especially multicellular organisms exhibit a structuring and maintenance ability beyond human design, which naturally leads to a special scientific interest in the biological building process that creates these organisms. The growth of an organism starts with a single fertilized egg cell which transforms through a development of concerted cell actions (such as division, signaling, etc.) into a mature and robustly functional assembly of cells. Embryogeny is the pre-natal part of that developmental process where a fertilized egg cell undergoes multiple divisions until an organism with spatially and functionally organized tissues and organs is created. Mimicking this process has been supposed to be the key to engineering complex artifacts [1], [2]. Building and maintaining functional artifacts with high complexity and robustness is a challenge engineers face throughout all fields of application. Specific approaches, such as using modular architectures and redundancy are suitable only up to a certain level of functionality. Therefore, many developmental approaches toward creating artificial systems have been proposed recently [3]–[14].

The implemented developmental mechanisms of such systems are generally based on an abstraction of observed principles in biology and can be divided into two main categories: phenotypic mechanisms and genetic control mechanisms: Phenotypic mechanisms are those parts of the models that are used to represent the developing shape or behavior. For example, they are the implementation of cells and cellular behaviors, such as division, adhesion, simulated physics; all kinds of non-signaling cellular interactions in general. Control mechanisms are the analog of the DNA and its signaling proteins in biology, i.e., the way a regulatory network is realized, which evolution acts directly on by changing weights and connections. In both domains, choosing the right abstraction level is difficult and clearly depends on the purpose of the resulting system. For example, simulating biological phenotypic mechanisms such as polarity and chemotaxis can yield a system with the ability to grow functional shapes [15] but does not per se imply predictive power for the evolution of development of biological organisms. Simulated developmental mechanisms are usually chosen very specifically, carefully taking into account other, already existing system features and the desired system behavior. As a result, most scientific findings from proposed models do not generalize easily.

This paper focuses on the investigation of a novel control mechanism for artificial embryogeny models. Most of the proposed models [3]–[14] employ a control mechanism of cellular growth via artificial gene regulatory networks (GRNs) that abstract biological gene regulatory networks using discrete or continuous formulations, and most implementations are unique. The uniqueness of the approaches results from the fact that no system has so far been shown to be superior to any other approach for a wide range of applications. Apart from implementations of artificial gene regulatory networks, control mechanisms are sometimes simulated by random boolean networks, multi-layer perceptrons, or continuous time recurrent neural networks. All of these approaches have in common that they create a nonlinear system where certain ‘output nodes’ are used to control development and ‘input nodes’ are carefully initialized to trigger dynamics or receive continuous environmental signals. Obviously, the implementation of such a system influences the way a graph change results in a change in system dynamics; but in general it seems that evolving networks to get desired dynamics is non-trivial [16], [17]. Small changes in network weights and structure do in most cases change the system phase space – and resulting development – unpredictably; sometimes to a great extent, sometimes not at all [18]. Although this fact can in some cases be beneficial for evolvability [19], it renders analysis of evolutionary steps in graph based embryogenies difficult.

Therefore, we suggest an abstract evolutionary embryogeny system based on vector field editing, where the use of a graph structure to build a nonlinear system is omitted in favor of evolving a system phase space directly. Note that the phase space represents the space of all possible states of a system. Hence, phase space is sometimes referred to as state space. We prefer to use the term phase space for its unambiguity [20], [21]. Our approach shifts the use of a system's phase space from pure visualization of its characteristics (e.g. [22]) to an encoding of its features. The advantage of this approach is that a phase space is a common feature of all dynamic systems, independent of their implementation. We expect scientific findings to be transferable to other systems with less effort than in previous models. Furthermore, the effects of evolutionary change can be visualized and easily understood, and features of graph represented systems, such as different kinds of attractor dynamics in the system phase space, are still available. Direct modification of the phase space can lead to more predictable changes in system dynamics. Thus, we can successfully evolve development toward desired patterns of cellular differentiation using standard evolution strategies [23]. By contrast, graph based approaches seem to require special attention toward evolutionary operators and environmental cues for similar tasks [24], [25].

This paper is structured as follows: We will first describe how a gene regulatory network can be replaced by its phase space. Then, we will introduce vector field editing, a method for directly changing features of phase spaces, and specify its usage in vector field embryogeny. Further on, we will show how a spatial hierarchy can be added to the framework, and how explicit evolution of allometric changes can be investigated. We follow the definition of allometry from a developmental biology perspective: Allometry is “[…] a shift in the growth rates of different parts of the organism relative to one another” [26]. Results from experiments for cellular differentiation using vector field embryogeny with and without hierarchy and direct evolution of allometry are presented, and compared to a GRN based method. We conclude with a discussion of these results.

## Materials and Methods

### From Gene Regulation to System Phase Space

Biological development is a process based on proteins; these products of expressed genes constitute both building material and control for creating an adult individual. We are interested in the control aspect: some proteins, called Transcription Factors, possess the ability to regulate the expression of genes by binding to the respective promoter regions on the DNA and thereby influencing their transcription processes. In this way, mutual interaction between genes occurs by means of their products, which eventually results in complex gene regulatory networks. GRNs are nonlinear systems that create complex patterns of gene activation. Nonlinear systems can typically be characterized by the following features [20]:

1. multiple isolated equilibria
2. limit cycles
3. subharmonic, harmonic, or almost periodic oscillations
4. chaos
5. multiple modes of behavior

Note that according to Khalil [20], *behavior* in item 5 refers to the set of dynamical features given in items one to four. Items 1 and 2 are abundant in the dynamics of gene regulatory networks. For example, multiple isolated equilibria can account for cell differentiation in biological organisms [27], and many different inter- and intra-cellular processes are represented by limit cycles [28], where probably the most prominent representatives are the circadian rhythms [29], [30]. Item 5 is the most prominent feature of life; the ability to adapt to different external conditions by switching between modes of operation can be found in virtually all organisms. Recently, it has been observed how a biological GRN dynamically changes its modes of operation, when environmental conditions are altered [31]. Items 3 and 4 are observed in computational models for biological GRNs [32]. Under certain conditions, simulated circadian clock genes exhibit chaotic and birhythmic behavior. However, it is argued that the smallness of the parameter range in which this occurs makes it unlikely to occur in biology. Also, known arrhythmic biological mutants of the circadian clocks seem to result from a severe structural change in the underlying network, rather than from normal mode of operation under certain environmental conditions [33].

In this light, the dynamic behavior of GRNs seems to account for the flexibility and robustness of biological organisms. Therefore, the most common approach to realize an artificial system with these features is to model the interplay between a number of genes to create regulatory networks. The natural representation of these networks is a directed graph. Each node of such a graph represents a state variable of the system, and the links indicate modes of interaction between nodes with connection weights and more or less complex activation functions. Standard approaches toward evolving these networks are based on evolving both, structure and weights of the networks (e.g. [34]).

**Figure 1:**
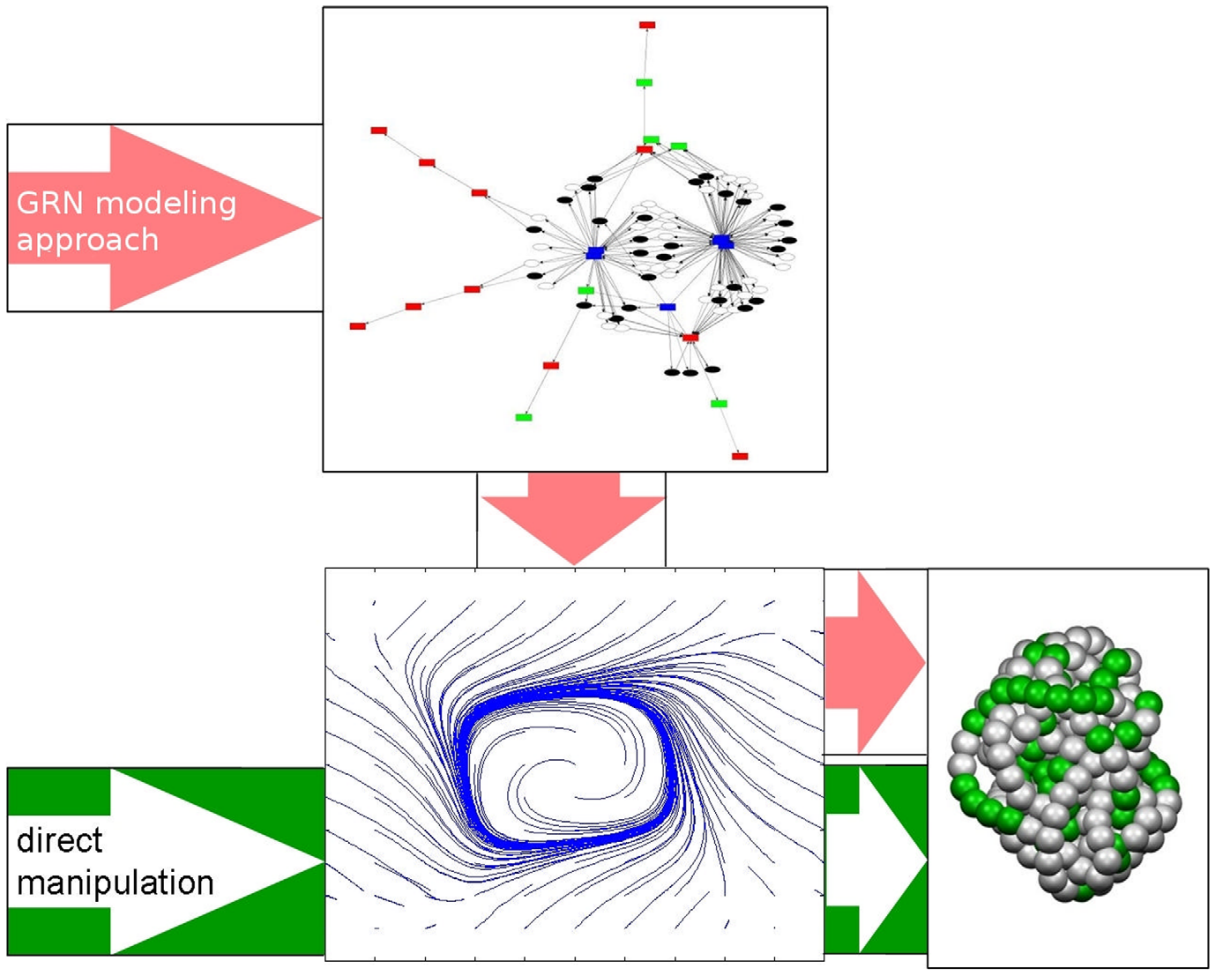
Two different approaches toward evolving control of development. The usual approach for evolving developmental processes consists of manipulating a regulatory network, which then creates dynamical system properties that control the developmental process (upper arrow). The approach presented here omits the network representation by directly manipulating the system phase space, i.e., the dynamic behavior of the system, to evolve a control for development (lower arrow).

In this contribution, we propose to shift evolutionary focus from the structure and weights of the network to the dynamics that such a network would create, i.e., to its system phase space. Figure 1 illustrates our approach: We enable mutation operators to directly create and shape the system phase space (direct manipulation), instead of doing so indirectly via graph manipulation. This allows a more causal relationship between mutation and resulting changes in system dynamics. Direct shaping of the phase space is inspired by a method known as vector field editing [35] and will be described in the following.

### Direct Manipulation of the Phase Space

In computer graphics, the vector field editing method is used for creating texture alignments and extracting analytical information about given graphical representations of vector fields [35]–[37]. To be able to apply this method to regulatory systems for artificial embryogeny, the formulation of an artificial developmental system must be viewed in an abstract way. The following considerations are presented using a two-dimensional version of the system for clarity and visualization purposes. Note that the method extends to D dimensions by applying the respective D-dimensional geometrical operations.

**Figure 2:**
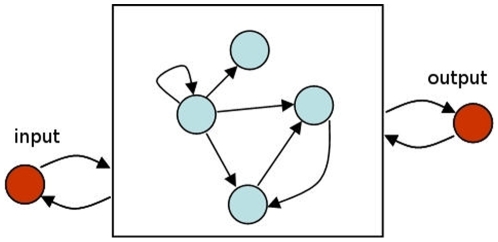
Schematic representation of a GRN with two observable system variables, e.g. one input and one output. Observable variables have a dynamic behavior depending on the GRN they are attached to.

**Figure 3:**
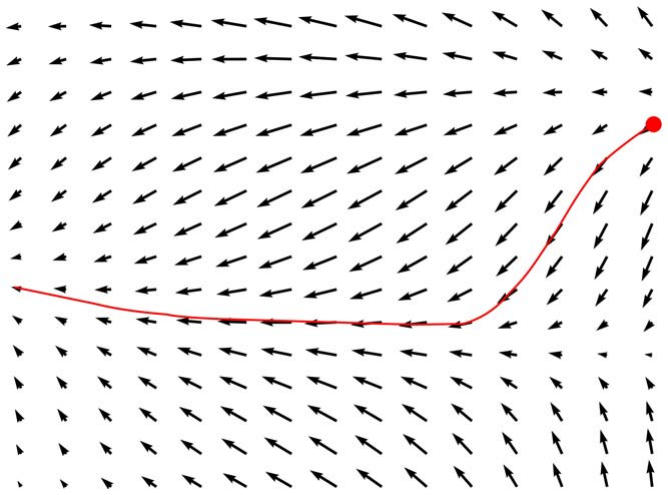
A two dimensional vector field can be interpreted as system phase space. The vector field gives the magnitude and angle of change of the system at every system state. A possible initial system state and the system trajectory which would result from the vector field are highlighted.

Consider an arbitrary simulated GRN inside a cell, with two genes of interest (Figure 2). We denote the state (i.e., activation level) of these two genes by 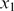 and 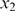 respectively, and together as the vector 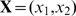. The temporal behavior of any deterministic simulation of a regulatory network containing these two genes can now be described with respect to 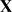 by the differential equation 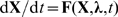, where **F** is a vector field and 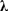 is a vector of parameters. The time dependency of **F** can result from different external influences: For example, a change in environmental conditions could be sensed by the cell and induce a different mode of operation, or a communication signal, such as a diffusing agent from neighboring cells, could alter the dynamics of a cell's GRN. Investigating these alterations of phase spaces during development is an exciting task. However, in this paper, we will focus on isolated cells in constant environmental conditions, such that 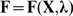. Hence, 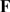 describes a time independent, two dimensional vector for each system state **X**, which represents the direction and magnitude of change in time, whenever the system reaches the state **X**. An example vector field and a possible resulting system trajectory are given in Figure 3. This kind of representation is known as the phase space plot of a system [21].

**Figure 4:**
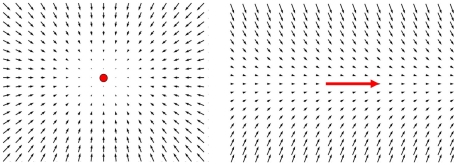
Vector field embryogeny relies on basic field elements. Two basic field elements are employed: a singular element is depicted on the left panel, and a regular element (attachment element) is depicted on the right panel. Point and arrow mark the center and center line of the elements respectively.

Vector field editing relies on creating and changing a vector field by superposition and adaptation of basic field elements 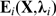. The vector field for any system state 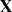 is then given by the superposition of these elements:
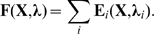
(1)To employ vector field editing for control of artificial development, we need to define basic field elements that are suitable to create a desired system phase space. Typical elements are proposed in [35] and [36] and can be grouped into singular elements and regular elements. Singular elements are those which create a singularity in the vector field (i.e., a source or a sink) while regular elements do not contain a singularity in their description, and thus generally change the vector field without creating singularities. Two examples are depicted in Figure 4.

In our framework, we adopt the regular element formulation given in [35] and use a simplified version of singular elements. The regular element we use is called attachment element and is depicted in the right panel of Figure 4. It creates a flow of surrounding system states toward an attachment line at the center of the phase space. The mathematical formulation to create such an element, where the attachment line is oriented along an arbitrary angle 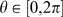 is given by
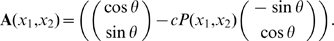
(2)Here, 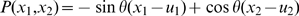 and 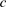 is a parameter describing the speed with which the flow is attracted to the line and 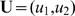 is the center position of the element. Note that for negative 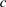, system states will diverge from the line instead of converging to it. To spatially limit the element's influence for superposition, this attachment element is multiplied by a Gaussian kernel 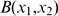 of width 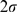 and center **U**: 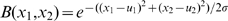. Therefore, the complete formulation of the attachment element is given by
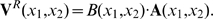
(3)We create a singular element by applying
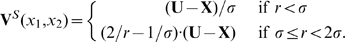
(4)The variable 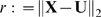 describes the distance of the system state **X** to the center **U** of the singular element. The width of the element is denoted by 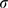. Formulation (4) is a coarse piecewise linear approximation of 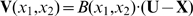. We use it, since it is more efficient in computer simulations.

A superposition of 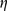 field elements, each weighted by a factor 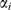, yields an arbitrarily complex vector field, which can be interpreted as system phase space:
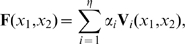
(5)where 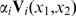 corresponds to 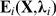 in Equation (1), with 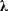 consisting of all 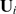,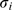,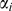 of all field elements, and additionally 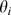 and 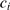 of the regular elements. Thus, the vector field described in Equation (5) constitutes the right hand side of the differential equation
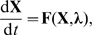
(6)which is integrated from 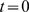 to 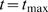 to yield a trajectory of the dynamic system.

### Experimental Setup

#### The general setup

For our experiments, we set up a phase space model in three dimensions, 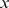, 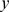 and 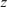, constrained to the interval 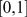 in each dimension. Thus, 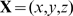 in Equation (1). This would correspond to a GRN where the state of three genes is observable during developmental time.

We then perform the following steps:

1. Determine initial states of these three variables for development (in a biological context, initial values may result from environmental signals or a maternal gradient).
2. Create a phase space in three dimensions, i.e., choose 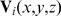.
3. Use an evolution strategy to mutate the parameters 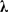 and thereby change the vector field representation of the phase space 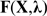.
4. For individual 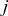, use the differential equation 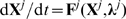 to create time courses of the corresponding three variables to control its development.
5. Use the evolution strategy to select fit individuals for reproduction and repeat steps 1 to 5 until a stop criterion is met.

To investigate cellular differentiation, the system state **X** is interpreted as the expression level of three genes in a certain cell of an individual. The cells that belong to the same individual share the same phase space, but have different initializations of 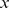 and 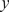. We define 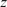 to correspond to the cell type and initialize it at 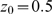 for all cells, representing a non-differentiated state. The cell's environmental information is encoded in 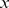 and 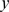 and can be interpreted as maternal factors, similar to those found in the early Drosophila embryo [38]. Cells do not divide or interact; note however that both mechanisms would be possible to include in the framework (see Discussion). For visualization, cells are positioned on a 2D lattice, where the coordinate of a cell is chosen according to its initial state of the genes x and y. Two different resolutions are used for experiments: 2×2 and 4×4 cells. Therefore, 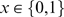 and 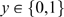, or 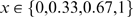 and 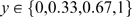 for the respective experiments.

The phase space of an individual is evolved by changing the key parameters of a fixed number of field elements. These key parameters for singular elements are 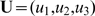, 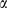, and 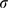. 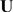 represents the position of the element in 3D space, 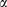 is its strength and 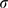 its width (see field element description above). For an attachment element, three additional parameters are encoded: 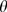, 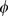, and 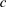. 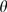 and 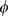 are the two angles describing the direction of the element in 3D space, and 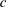 is the relative speed of attachment (see Equation (2)). The resulting system equations are solved for each cell by a Runge-Kutta method of order 4. The maximum simulation time is set to 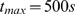, with a step width of 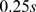 and 8 sub-iterations per step. We expect system states to have reached a stable state before the simulation time reaches 500 seconds. However, if this is not the case, solutions are not penalized. In fact, our simulation of allometry relies on system states being in a transient state at 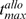 (see Experiments 2 and 4 for details). Simulation is terminated when either the maximum time 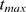 is exceeded or when the system state does not vary more than 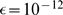 in two consecutive steps. A standard evolution strategy [23] is employed, with population sizes of 15 and 100 for parent and offspring population respectively, with a single strategy parameter with step size adaptation. A more sophisticated evolution strategy could be applied ([39] gives a comprehensive overview), however, the standard version is very robust and its performance is sufficient for our purpose. The initial strategy parameter is chosen to be 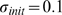. The fitness 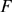 is calculated by taking the squared distance between the cell types of the 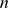 cells belonging to an individual after development, and a given target vector 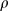: 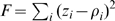. Therefore, the task is a minimization task, and optimal fitness is reached if 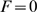. Note that for the experiments presented in the following, the maximum value for 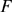 is the number of simulated trajectories, i.e., 4 for the 2×2 and 16 for the 4×4 runs since both, 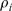 and 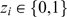. Twenty evolutionary runs are performed per experiment.

Note also, that the adaptation of the framework to a specific research problem is basically similar to setting up a graph based method. The fundamental difference lies in the evolution of the system: while both approaches possess the ability to represent complex phase spaces, vector field embryogeny creates a more causal relation between mutation strength and phase space change. For the evolution strategy we use, which employs normally distributed mutations, we can expect the aforementioned causality to improve evolvability. Apart from evolvability, the simplicity of a representation is important for analysis and understanding. We believe that a spatial illustration in up to three dimensions of dynamic system properties, and especially of mutational changes in dynamics, is more intuitive than inferring system behavior changes from graph structure changes. Note that this does not necessarily decouple our observations from regulatory networks or biology: we merely investigate evolution of dynamical behavior on a systemic level, where both, regulatory mechanisms and evolutionary processes, are modeled abstractly, and thereby provide a different point of view on their respective biological counterparts.

For comparison, we employ a GRN model for the same evolutionary tasks. The model we choose as an example for GRN based approaches is described in detail in [34]. Briefly, in this model, cellular activity is controlled by a genome stored inside a virtual DNA (vDNA), of which an identical copy is available for translation to all cells in an individual. This genome consists of regulatory subunits (RUs) and structural subunits (SUs), which are initially lined up in a random order. A functional unit of this vDNA, called a gene, is composed of a group of SUs and its preceding RUs. The SUs encode rules for the production of transcription factors, while the RUs determine whether a gene is active or not. The transcription factors encoded in a gene will be produced only if the gene is active. Both RUs and SUs are represented by a set of double precision values which are evolved using the evolution strategy. During simulation of dynamics, the vDNA is translated to produce transcription factor concentrations. The rate of production is influenced by the RUs, which evaluate the concentration of other transcription factors to determine an activation value for a gene with which the production rates of gene products are scaled. Thus, genetic interaction yields a regulatory network.

As indicated in the introduction, it is difficult to define a reference GRN model. Therefore, our choice of model is primarily based on the experience of the authors using it, and not on an assumed superiority to other GRN based approaches. However, the model has several key characteristics that can be found in most evolutionary development models, and therefore renders it a good candidate for comparison. These characteristics are: ability to mutate structure and interaction strength of the genes, gene duplication and transposition for complexification and modularization, possibility of feedback loops and signal decay, and dynamical operon structure (i.e., several structural units can be controlled by the same regulatory units). The model has been shown to be suitable for several different evolutionary development tasks ([15], [34], [40]).

For the GRN model, we choose the following experimental setup, and also perform 20 runs per experiment (see [34] for details): A genome size of 20 initial regulatory units and 20 initial structural units is employed. These are empirical values which we have used in various simulations and consider suitable for the given task. Note that through duplication, the number of units can be adapted by the evolutionary process. Two constant pre-diffused gradients with linear distributions along the two axes are defined, which represent the x- and y-coordinate of the experiment. Cells are positioned on the x-y-plane in the same manner as in the phase space experiments. Cells do not communicate, i.e., signals do not diffuse in space. The concentration of the first genetically created transcription factor, i.e., the activity of one gene, in each cell is used for fitness evaluation, following the fitness function given above. This setup creates a task for the GRN based system, which is comparable to that of the vector field embryogeny. From experience with the GRN based system we know that good solutions are often lost through mutations. Also, the standard selection pressure is usually too high and yields early convergence to local optima. Therefore, it is necessary to slightly deviate from the standard evolution strategy employed in the vector field embryogeny framework, to achieve comparable results: Firstly, three elitists [23] are employed in the evolution, i.e., the three best individuals of a generation are carried over to the next generation without mutation. Secondly, parents population size is increased to 40 to reduce selection pressure. Finally, the 4×4 cells task is run for 200 instead of 100 generations.

#### The allometry setup

Evolutionary changes in biological development are either spatial or temporal [26]. The temporal aspect has not been given explicit attention in the artificial development community, apart from defining intermediate or final stages of developmental processes, after a defined number of time steps [5]. It has even been argued that the temporal aspect can be neglected in developmental systems by replacing the developmental mapping with a CPPN (Compositional Pattern Producing Network) [41], a feed forward artificial neural network like structure with special activation functions. In biology, the significance of developmental time for evolution has long been recognized and widely studied. Time in self-organization processes, particularly in spatial pattern formation, is known to play an important role, see for example [42], [43]. We will briefly describe the biological point of view on a system feature called allometry, and elaborate its implications for vector field embryogeny.

Examples of time-dependent processes that are considered to be advantageous for the progress of evolution in biology are: allometry and heterochrony (see e.g. [44]–[47]). We are interested in the effect of allometry and will therefore focus on its description. Allometry occurs when different parts of an organism grow at different rates in distinct species [26]. An example can be borrowed from Wolpert [48]: the central toe of a horse grows at a rate 1.4 times that of lateral toes. This allowed for evolutionary adaptation by formation of the typical shape of the horse hoof, originating from ancestral multi-toe feet. In this example, the size of a toe can be seen abstractly as a variable in the phase space of hoof development. In this light, toe development can be seen as a transient process. At a certain point in developmental time, the outcome of this process (i.e., the relative size of the toes) is a result of the evolved rates of change of the toe-size variable, achieved by a scaling of the relative speed of system dynamics of the toes. Therefore, allometry can be seen as a means to evolutionarily change the transient behavior of several microscopic parts of a system to influence its final macroscopic shape. An interesting question for artificial embryogeny frameworks would be: What are the consequences of allowing for direct evolution of the rate of cellular processes?

The framework described above allows us to investigate this question. We can include allometry by evolving a speed factor for every cell-state 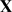, which scales the speed of the system state on its trajectory through the 3D phase space. Thus, in different individuals, 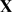 could reach different states in finite time, even if the phase space would be exactly the same. In practice, we encode 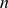 additional allometry variables, 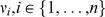, which are speed factors of the 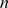 cells in an individual. These variables lie in the interval 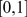, and are used to scale 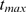 for the integration of Equation (6), i.e., 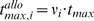 for the 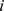-th cell, representing cellular phase space speed.

#### The spatial hierarchy setup

During biological embryogeny, organisms go through a phase of hierarchical structuring [26]. A spatial hierarchy develops over time, such that early signals in the embryo create a coarse structuring, while later signals are used to create more and more details of the final morphology. Doursat [49] has used such a hierarchy to create spatial differentiation during an artificial growth process. We show that it is possible to integrate a similar mechanism in vector field embryogeny.

**Figure 5:**
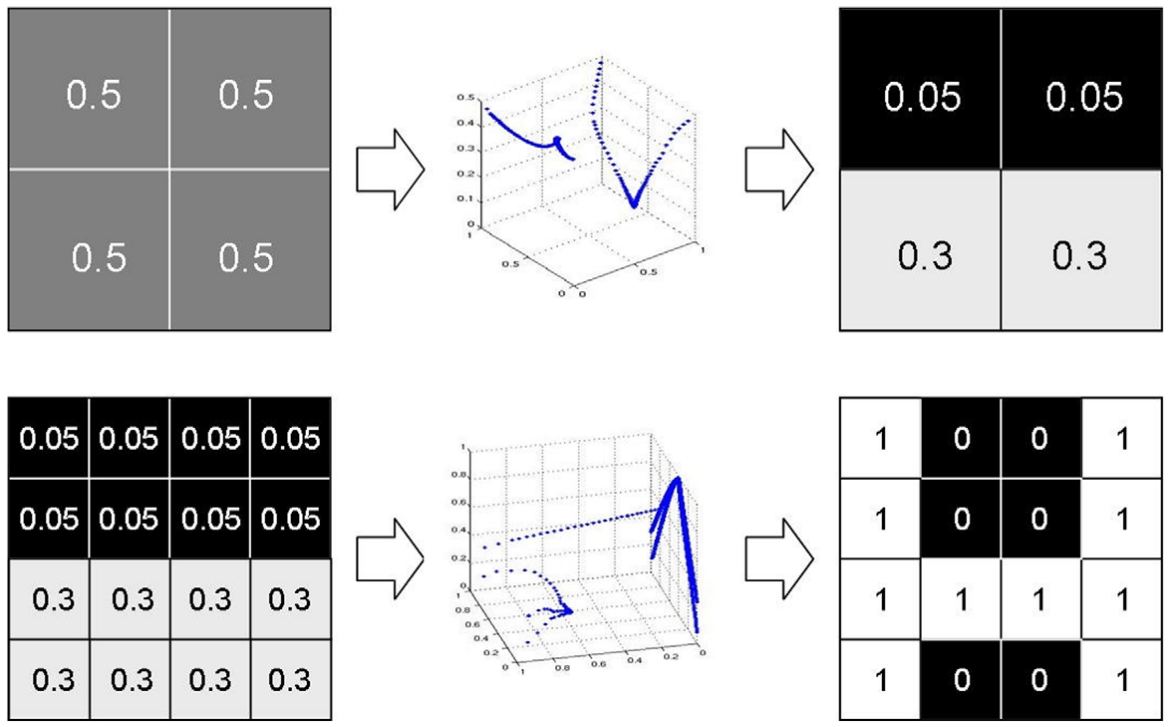
Two hierarchical levels of development. Upper panel: four cells are initialized with cell type 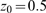 and 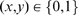. In the 2D representations, 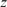-positions are mapped onto the 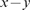 plane according to the cells' initial states (i.e., the lower left square gives the 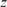-value for a cell with 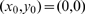, the lower right square gives the 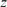-value for a cell with 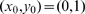 and so on). State trajectories in the phase space are given. After the system states of the first stage have reached their final position in phase space, their 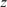-values mark the initial cell type of the four respective cells in the second, fine grained level (lower panel). These are initialized with 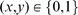 again for each coarse level cell. Note the symmetric coordinates in the second level (see also Figure 7).

Consider our example above, with a three dimensional phase space 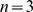, 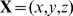, where 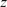 gives the cell type and 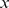 and 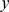 the variables which carry initial conditions. Let us assume that we simulate four cells of an individual such that four trajectories, 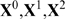 and 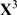 will be simulated in the phase space, with initial conditions 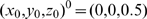, 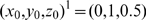, 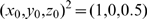 and 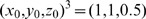, i.e., the four corners of the 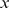-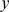-plane at 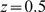. The common phase space represents the common genetic control of the cells of an individual. The implementation of spatial hierarchy in this three dimensional vector field embryogeny can be based on a subsequent subdivision of initial system positions on the 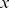-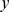-plane, where each system state **X** has the ability to divide into four ‘daughter’-system states to constitute the next hierarchical stage. Thus, if the initial stage consists of four initial system states, the second stage will contain 16 and the n-th stage 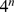 system trajectories. Each hierarchical stage has its own phase space, with own evolving field elements. When a system state is subdivided, its daughters are initialized such that the ‘cell type’ variable, i.e., their 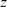 positions in phase space, are equal to the final ‘cell type’ of the mother cell, while 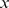 and 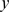 are chosen to be the corners of the 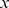-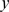-plane again in the phase space of the respective hierarchical stage (see Figure 5). Since one hierarchical stage has only one phase space, all cells belonging to one stage share the same phase space. In approaches using multiple stages of hierarchy, this allows a reduction in parameters: if a field element can be described by 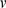 variables, and 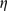 elements are used in each of the 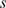 stages, the total number of variables to describe one individual solution is 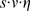, while the number of cells that can be described with this setup amounts to 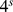.

**Figure 6:**
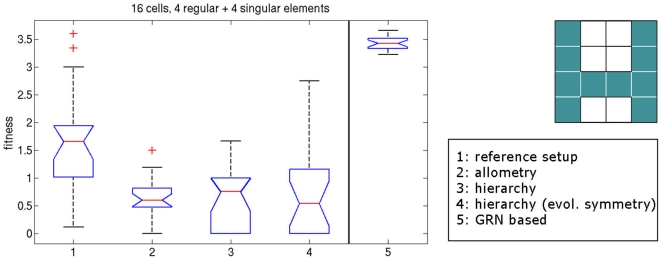
Results from the differentiation experiment with 16 cells. The ‘H’-target pattern and the results from the different setups are presented. The results of the reference setup, the allometry setup and two hierarchy setups (pre-defined and evolving weak symmetry) are depicted, as well as the result of the respective GRN based approach.

**Figure 7:**
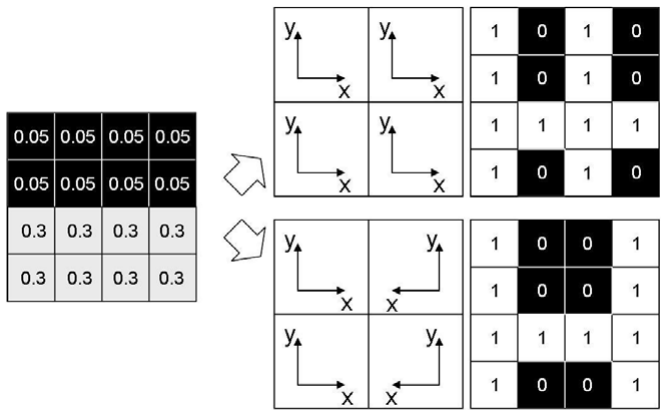
Non-symmetrical and symmetrical setup of the experiments. Explicitly changing the coordinates of the initial state of a fine grained level allows exploitation of symmetry. The upper panel shows the resulting final cell-type distribution from the experiment presented in Figure 5, if the weak symmetry constraint is not employed. The lower panel shows the same result using a weak symmetry constraint.

This setup also allows an explicit integration of weak symmetry constraints, i.e., for symmetry with variation. The right panel of Figure 6 shows an example of a lateral symmetric target pattern. The left half of the target can be reproduced by the same mechanism which creates the right half, if the underlying coordinate system is mirrored. Let the initialization of the coarse stage system be 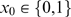 and 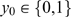, and 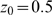, i.e., one cell in each corner of the 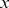-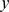-plane, each with the same type, as depicted in Figure 5. The first stage of development yields two pairs of cells, each consisting of system states that reach the same type 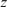 during development (Figure 5, upper panel on the right). Accordingly, the four trajectories of the next stage for each cell pair will start at the same height 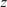. To illustrate the symmetry in the resulting cell states (Figure 5, lower panel on the right), let us consider Figure 7: since the 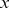 and 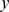 coordinates are initialized equally, the fine grained solutions would be identical for those cells that have reached equal 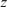 during the first stage (Figure 7 upper panel). If the initial 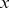 coordinates are mirrored however, the solution will be symmetrical (Figure 7 lower right panel and Figure 5, lower panel). Note that perfect symmetry is only facilitated by this formulation, but not enforced: if system states in the first hierarchical stage converge to 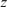 values that differ between left and right, the initial states for the second hierarchical stage are distinct and can therefore yield different trajectories with different end points, which eventually results in non-symmetric patterns. Hence, in this setup we adopt the term weak symmetry constraints.

## Results

### Experiment 1: Cellular Differentiation

**Figure 8:**
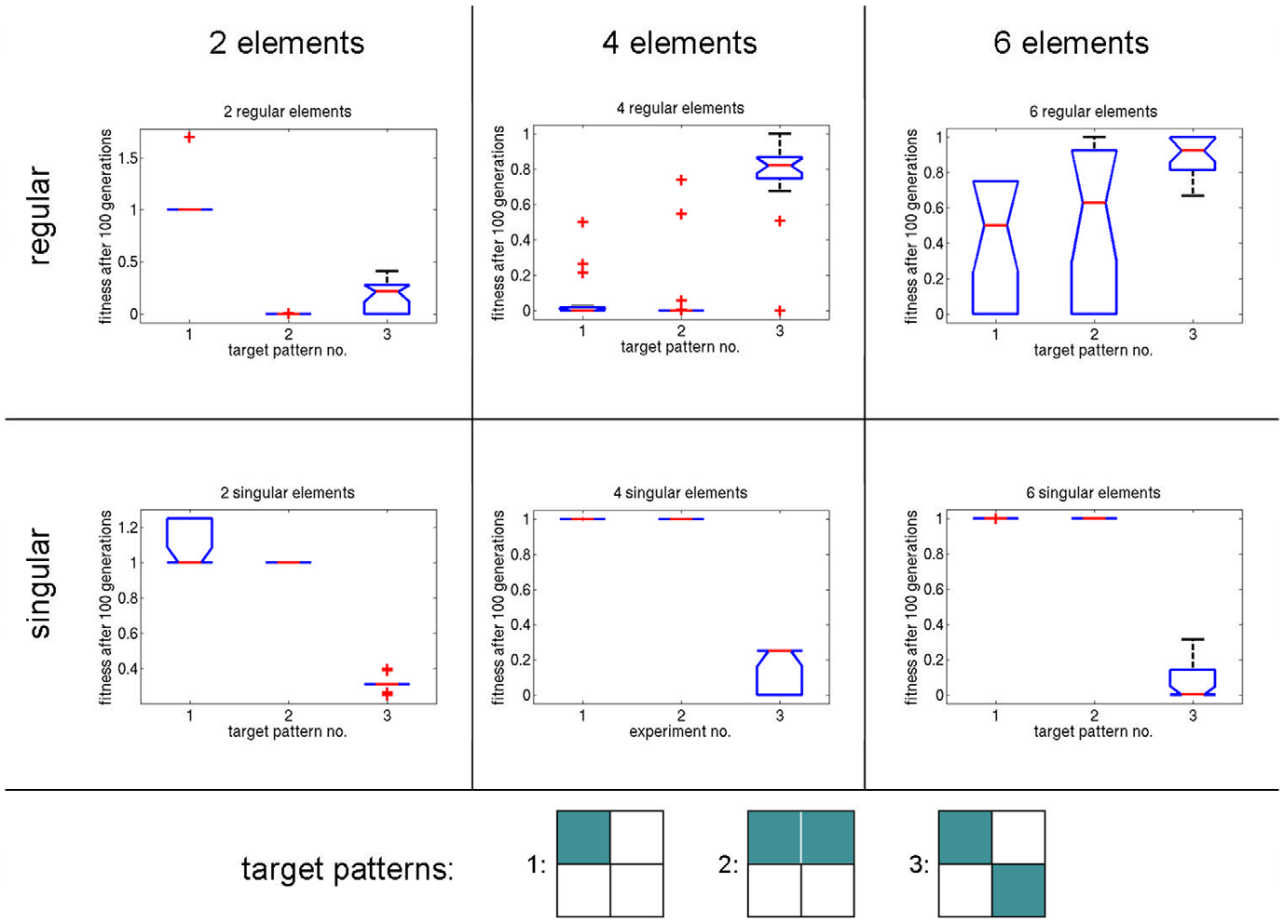
Results from the differentiation experiment, summarized in 6 plots. Experimental setups with different numbers (two, four, and six) and types (singular and regular) of basic field elements are compared. The experiments were conducted using three different target patterns: ‘one point’ (1), ‘half’ (2), and ‘xor’ (3).

The first experiments show the feasibility of vector field embryogeny to evolve cellular differentiation. The possibility to generate an arbitrary cellular distribution in a 2×2 cell grid is investigated. To this end, three target patterns are defined: ‘one point’, ‘half’, and ‘xor’ (see Figure 8). Note that for the trivial solution of 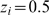, i.e., no movement in phase space, a fitness value of 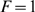 would be the result for all targets. We denote fitness values below 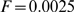 as optimal. The experiments investigate the influence of field element type and field element number on the evolvability of the system: we first perform evolutionary runs that employ singular elements or regular elements exclusively. The number of elements in these experiments is varied between 2 and 6.

Figure 8 gives the results of the experiment after 100 evolutionary generations. For the target patterns ‘one point’ and ‘half’, using 4 regular elements shows good performance, while for the target pattern ‘xor’, using 6 singular elements yields the best results. Interestingly, using singular elements only leads to early convergence of the ‘one point’ and ‘half’ runs, while using regular elements yields suboptimal performance for the ‘xor’ run. Generally, using 6 regular elements yields evolutionary runs without convergence after 100 generations, which can be seen in the variance of the fitnesses. It seems that the exclusive strategies with one type of field element only, are suitable for certain characteristic differentiation targets only.

**Figure 9:**
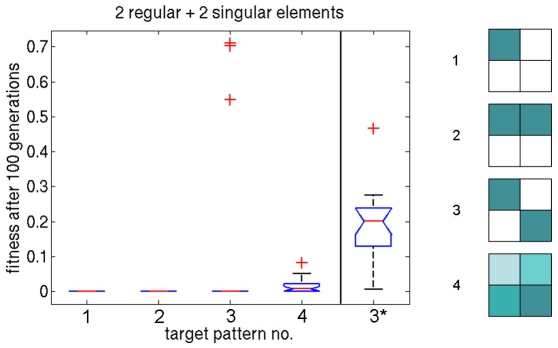
The concluding experiment of the differentiation task. Two regular elements and two singular elements are combined, and applied to the four target patterns: ‘one point’ (1), ‘half’ (2), ‘xor’ (3), and ‘random’ (4). For comparison, the performance of a GRN based approach for the ‘xor’ target pattern is shown on the rightmost panel (3*) with similar evolutionary setup (see text for details).

**Figure 10:**
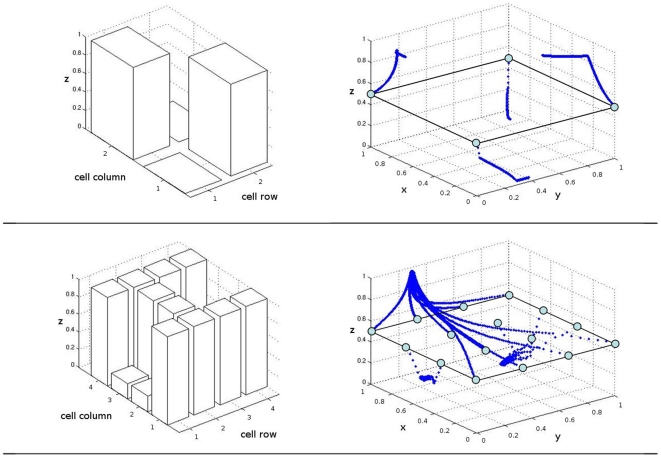
Final cell-type distributions of successful individuals. Upper panel: The ‘xor’ pattern result and the corresponding evolved phase space trajectories for a run using two regular and two singular elements. Lower panel: The ‘H’ target pattern result of the best run using the reference setup, with the corresponding phase space trajectories.

Therefore, we create a setup with 4 field elements in total, where we combine two regular and two singular elements. Results are shown in Figure 9: A high fitness for all target patterns is achieved. The ‘one point’ and ‘half’ experiments are successful, while the ‘xor’ experiment has 3 outliers apart from all other runs reaching optimal solutions. In the rightmost column, results from the GRN-based approach toward the ‘xor’ target pattern are shown. Additionally, results from a random target pattern run, using the same setup are presented. Each evolutionary run has a target consisting of four values independently drawn from a uniform distribution in the interval 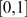. To visualize phase space trajectories, the upper panel of Figure 10 depicts trajectories of a successful individual of the ‘xor’ run and its final differentiation pattern.

These experiments show the feasibility of the vector field embryogeny approach to cellular differentiation tasks while on average, the GRN approach converges to lower quality solutions for the ‘xor’ target. The setup is now changed to a more complex task in a 4×4 grid where the ‘H’ target pattern is used (see Figure 6, upper right panel). Note again, that for the trivial solution of 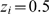, i.e., no movement in phase space, a fitness value of 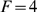 would be the result for all targets. We denote fitness values below 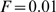 as optimal. In these experiments, the number of field elements is increased to 4 regular and 4 singular elements per experiment. Results are depicted in the leftmost column of the left panel of Figure 6. While no run reaches global optimum, the best individual's phenotype resembles the target and is shown in the lower panel of Figure 10, together with its trajectories in the phase space. In the following, we will refer to this experimental setup as the reference setup.

The rightmost panel of Figure 6 gives the performance of the GRN approach toward solving the same problem. Clearly, the GRN method is not able to generate the given target, despite the fact that twice the number of evolutionary generations are available. The mean fitness has a magnitude comparable to that of the two worst reference setup runs.

### Experiment 2: Evolving Differentiation with and without Allometry

To investigate whether allometry can have a positive effect on the evolution of phase spaces, we employ the allometry setup described above. Note that due to the initialization of the cells at 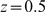, a target consisting of ones and zeros cannot be reached trivially by optimizing the allometry variables only, e.g. through setting 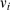 to 0 for some cells, and to 1 for the remaining ones.

**Figure 11:**
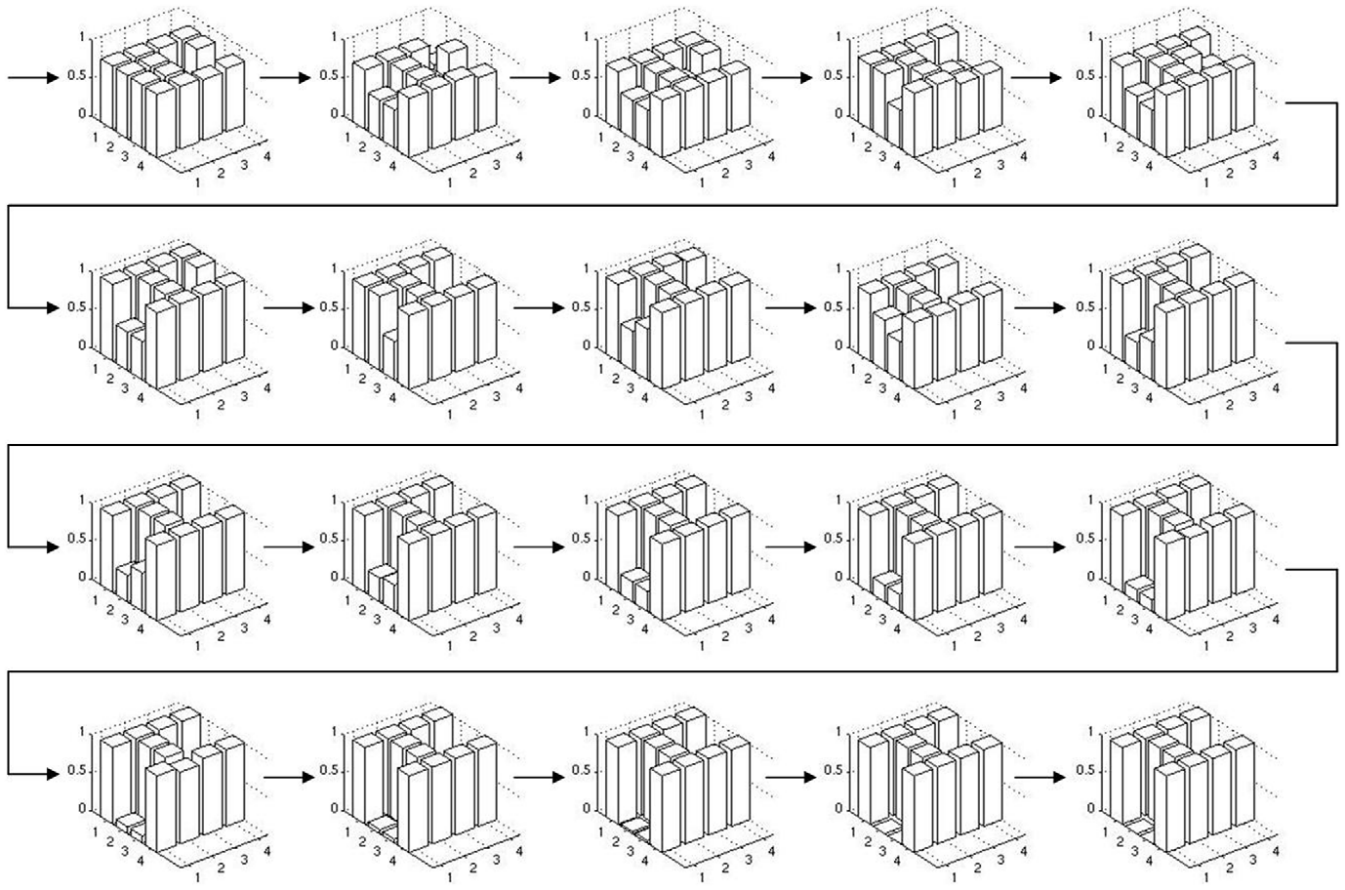
An evolutionary perspective on phenotypes. The best phenotypes throughout an evolutionary run of the allometry experiment are depicted for every fifth generation from generation five to 100. It is visible how evolution optimizes phenotypes toward the desired target shape.

**Figure 12:**
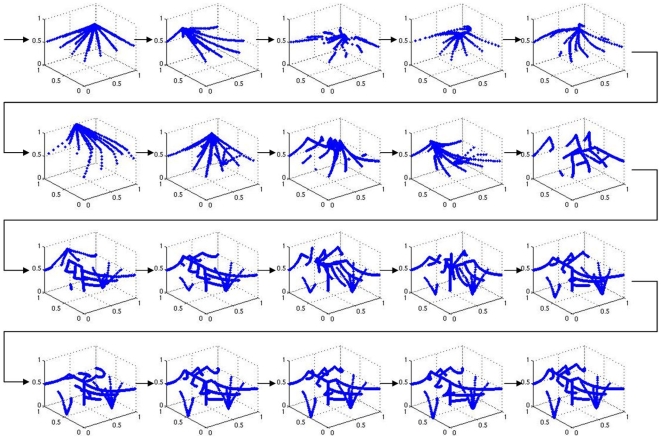
An evolutionary perspective on phase spaces. The phase space trajectories of the individuals given in Figure 11 are depicted. Both, the cellular differentiation toward attractors, as well as the changes of attractor positions can be traced throughout evolution.

**Figure 13:**
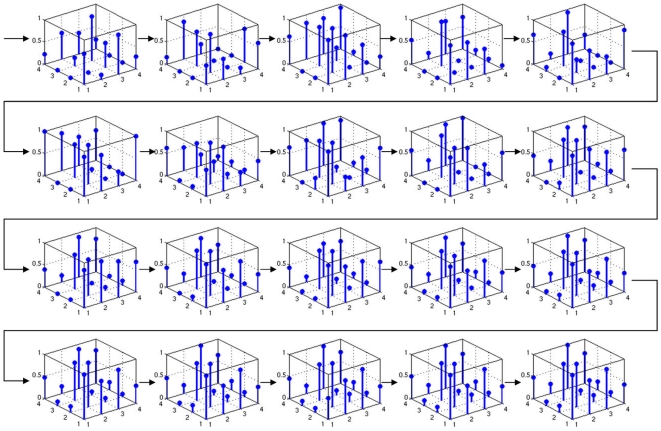
An evolutionary perspective on allometry. The allometry variables 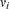 belonging to the individuals given in Figure 11 show a heterogeneity throughout evolution.

**Figure 14:**
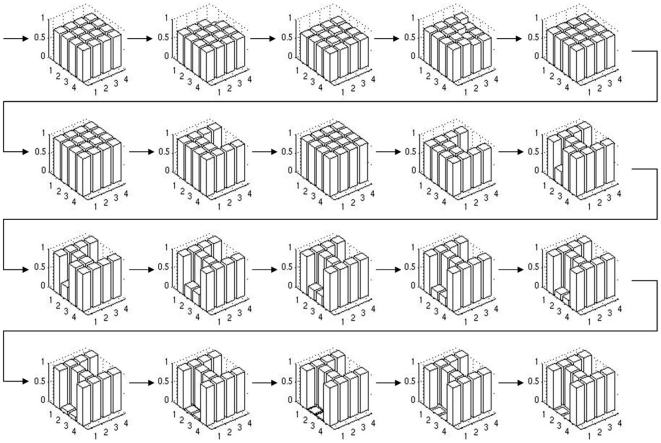
An evolutionary perspective on modified phenotypes. The phenotypes throughout evolution are depicted, where genotype is taken from the individuals given in Figure 11 and allometry is switched off. The final individual is allometry-independent after evolution.

Column 2 in the left panel of Figure 6 shows a significant increase in the performance of the system, with a mean fitness 0.55 and smaller variance than in the reference setup. For a thorough analysis, we now concentrate on the most successful run with allometry. We depict phenotypes for generations 5 to 100 in steps of 5 (Figure 11). The corresponding system trajectories are given in Figure 12. The distribution of the according allometry variables 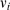 of the best individuals throughout evolution is given in Figure 13. The system seems to have one attractor for all points throughout the first generations. Around generation 35, a new, lower (i.e., 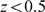) attractor is found. Prior to that, all distinct cell type- (i.e., z-) values were resulting from allometric scaling on the way to the upper attractor, i.e., by cells being in a transient state. After generation 35, the system settles for this configuration while optimizing the z-position of the new attractor. A third attractor is found around generation 50, which yields the basis vector field setup for the final solution. Until generation 100 is reached, the positions of these three attractors are optimized to yield a perfect solution. In the final individual, all cells have found suitable trajectories. Additionally, we investigate the role of allometry in the developmental process of the evolving individuals: we artificially switch off allometry by setting all 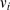 to 1 and repeat development for the best individuals of each generation throughout the evolutionary run (Figure 14). Interestingly, time plays no role for the development of an individual belonging to later generations. Indeed, after generation 60, the phenotypes of the original evolutionary run and the non-allometry run are the same (compare Figures 11 and 14). We investigated this feature in all 20 runs of the experiment and found that this holds only for the best run; all other runs produce individuals that depend on allometry. Therefore, the question remains whether the evolutionary success of the best run is directly linked to this feature.

### Experiment 3: Evolving Differentiation with a Two Stage Spatial Hierarchy

To investigate the influence of spatial hierarchy, we first switch off allometry. In addition, we allow for weak symmetrical boundary conditions (see Materials and Methods section) by choosing the coordinate system according to the lower panel on the right of Figure 7. The second hierarchy experiment uses a formulation that allows free evolution of symmetry, by encoding eight additional variables to be evolved. For each first stage cell, two of these variables (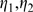) are used to determine whether the coordinate system for the respective second stage is flipped horizontally and vertically (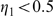 and 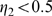, respectively). The number of field elements is set to 8 in total, i.e., 4 for the first and 4 for the second stage. A combination of two regular elements and two singular elements in each stage is used.

**Figure 15:**
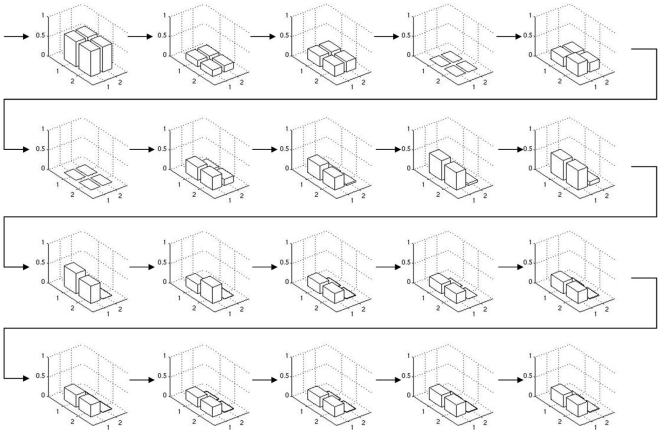
The evolution of the coarse stage phenotype in a 2-stage hierarchy experiment. Every fifth generation from generation five to 100 is depicted. See Figure 16 for the fine grained stage, note that both stages evolve simultaneously.

**Figure 16:**
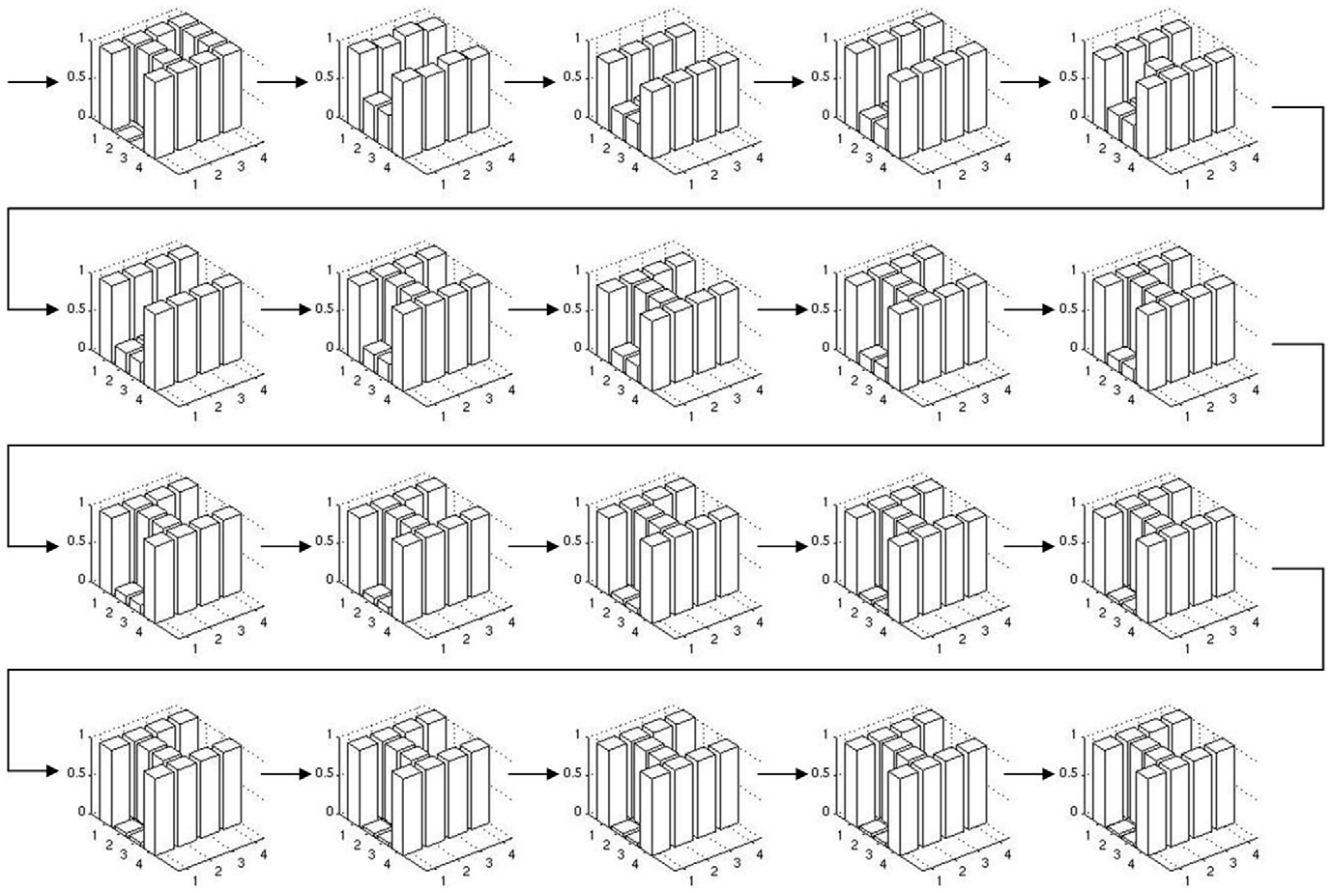
The evolution of the fine grained stage phenotype in a 2-stage hierarchy experiment. Every fifth generation from generation five to 100 is depicted. See Figure 15 for the coarse stage, note that both stages evolve simultaneously.

Both approaches perform significantly better than the reference setup, reaching a mean fitness of about 0.6 and 0.4 respectively (see Figure 6, panels 3 and 4). In Figures 15 and 16, we depict the evolution of the best individual's phenotypes coarse and fine grained stages in the symmetrical boundary conditions run, from generation 5 to 100 in steps of 5. It is visible how evolution finds symmetric solutions in the coarse stage and then uses this pre-structuring in the second stage to build a perfect solution to the target matching problem. Note that asymmetric solutions are possible even though the symmetry constraint is used (generation 25) and how a symmetric solution can still be reached in the second stage even if the first stage is not symmetric (generation 60).

### Experiment 4: Evolving Differentiation with Hierarchy and Allometry

The following experiments use a combination of hierarchy and allometry. We investigate three different experimental setups: using allometry on the first hierarchical stage only, using allometry on the second hierarchical stage only, and using allometry on both hierarchical stages. For these experiments, the weak symmetrical boundary conditions apply.

**Figure 17:**
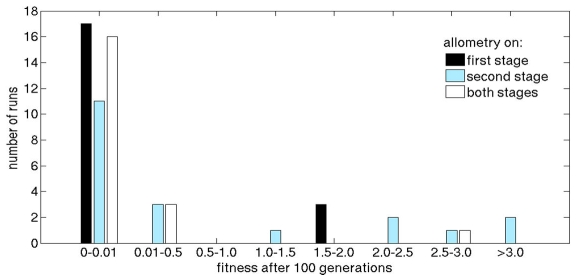
Results of experiments combining allometry and hierarchy. Allometry is employed on the first stage only (resulting mean fitness: 0.250), on the second stage only (resulting mean fitness: 0.882), and on both stages (resulting mean fitness: 0.178), respectively. The plot shows how many of the 20 evolutionary runs reached indicated fitnesses. Note that the first bin is scaled to a small size (0.0–0.01) to account for the high quality of the solutions.

The results for the three different setups are depicted in Figure 17. We can see that hierarchy combined with allometry on both stages reaches the optimal solution with only few outliers. Using allometry on the second stage only does not improve performance significantly. Interestingly, using allometry explicitly on the first stage gives exceptionally good results. Optimal solutions are found in 17 out of 20 runs.

## Discussion

We will first discuss the results of the experiments and conclude with a general discussion on the framework. The cellular differentiation experiments show the necessity of qualitatively different kinds of basic field elements (i.e., singular and regular elements) to achieve sufficient flexibility to solve the differentiation problems satisfactorily. The reference setup reaches a limit when it comes to the ‘H’ target pattern. Apart from investigating other basic field elements, many mechanisms could be used to augment the basic framework. We investigated allometry for the ‘H’ target pattern, and found that it improves evolutionary performance significantly. The question remains however, whether the allometry setup creates a ‘shortcut’ for the solution of the problem, i.e., that the better performance stems mostly from directly optimizing the 16 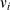-values and thereby rendering the evolution of the phase space trivial. One trivial (and sub-optimal) solution could be such, that the phase space consists of a single point attractor at 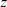 = 1, which attracts all cell states. If now 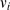 of the 6 cells in the interspace of the ‘H’ shape evolve to be 0, the interspace would remain at initialization level 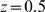. However, this would yield a fitness 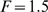, a value which is higher than the fitness reached in all but one evolutionary runs. Another seemingly simple solution would be a phase space that attracts all cells to a phase space trajectory which reaches 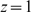 at one time and 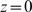 at another time, and then tuning 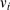 of all cells such that they stop at these two points. However, considering the basic elements we used, and the initial positioning of cell states, creating such a phase space would be extremely difficult, since the trajectories would have to cross their plane of initialization at 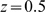.

The analysis of the trajectories throughout evolution shows nicely how the phase space evolves to accomplish the task. It is interesting to find a solution independent of allometry, although the evolutionary run has this feature enabled. Since allometry plays a role in the individuals in early generations, it has an effect on the course of evolution, yielding an evolutionary path to the optimal solution, which without allometry was not found in any of the reference evolutionary runs we have performed. Future work will analyze this influence in more detail.

Formulating the system hierarchically yields an insight into how a strategy might look like, with which to tackle more complex problems using vector field embryogeny. The weak symmetry constraints and evolving symmetry experiments have significantly improved performance for the given task, although the evolving symmetry runs yield a relatively large variance in quality. A remaining research question is the definition of the weak symmetry, especially when more than two stages are employed: should the symmetry constraint be inherited from the coarse stage to the next stage? Should it be redefined for each stage and each quadruple of cells? If so, how can we overcome the exponential increase in parameters to encode symmetry information?

Combining the hierarchical framework with allometry yields exceptionally good results. For allometry on the first stage, 85% of the runs converge to an optimal solution, for allometry on the second stage, only 55% converge to the optimum, and for allometry on both stages, 80% converge. Especially the case of using allometry on the first stage only is interesting, since the performance is not significantly different from employing allometry on both stages: it seems that if evolution has a more direct control of the first stage values, the second stage does not need that level of explicit control. If we use the same setup and evolve the four cell types of the first stage explicitly by direct coding (i.e., encoding 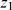 to 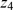 directly in the chromosome), all runs converge to the optimal solution (not shown). This finding motivates a setup where several hierarchical stages are employed, and the explicity of evolutionary control rises towards more coarse stages, such that e.g. in the four cell stage, a direct coding could be used, in the 16 cell stage a vector field with allometry, and in a subsequent, 64 cell stage a vector field only.

An interesting future research work for vector field editing in general would be to identify more useful basic elements, and assemble an element-library, possibly with grouping into different element classes for different kinds of problems. Also it would be interesting to research a complete field representation, i.e., a set of field elements that represent an arbitrary field, such that an assessable representation error exists if parts of the representation are neglected, in a way comparable to Fourier or Taylor series.

Throughout this contribution, we motivated vector field editing as an abstraction of biological GRNs. It is straightforward to map a network via its phase space onto a vector field. However, the opposite direction is difficult to accomplish: A given vector field in general can not easily be converted to a graph. This is mainly due to the fact that desired dynamics cannot be generated easily, e.g., by a superposition of graph-features. Therefore, the basic field elements of vector field editing are not to be seen as equivalent to network motifs [50]. Note that the search for functional sub-units of graphs that govern graph dynamics is still a debated topic [51].

Dynamics of GRNs account for many of the desirable features of organisms, such as robustness and flexibility during embryogenesis, which both make species evolvable. Many intricate regulatory interactions have been selected for in biology to create this evolvability. However, the exact underlying processes and driving forces are unknown. The concept of motifs has been successful to reveal structural coherence in the composition of biological regulatory networks. Still, the coupling between the embedding of a motif in a graph structure and the resulting changes of the dynamics remains unclear. The phase space approach suggested in this work concentrates on the more abstract level of the evolution of the dynamics of a regulatory system. It neglects its conceivable structural realization. This kind of explanatory level of dynamics combined with the analysis of graphs could enable us to understand the mapping between graph structure and phase space. We believe that this could hold the key to further our understanding of the evolution and function of biological regulatory networks.

To conclude, we have presented a novel approach toward evolving dynamic systems for the control of artificial embryogeny processes, which allows us to circumvent the problems generally associated with the evolution of graph based embryogenies. We have demonstrated the advantage of including advanced systemic features, such as hierarchy and allometry into the framework. Our experiments using the framework show that both hierarchy and allometry can be beneficial for increasing evolvability of a developmental system. A future work will be to more thoroughly compare vector field embryogeny against graph driven developmental systems, in terms of both, evolvability and computational effort. The novelty of the approach necessitates the investigation of a number of straight forward extensions to the system, e.g. the allometry variables 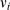 could be implemented using indirect coding, by representing them via spline or polynomial approximation. In this work, we have only considered static vector fields. When we think of a time dependent vector field during development, many parallels with biological systems can emerge; cellular communication for instance could be interpreted as a change in phase space for a certain cell, depending on its current state and the state of the surrounding cells. Also, the issue of complexification in evolution can be investigated in several ways; an obvious way to complexify a given phase space would consist of a gradual increase of its number of basic field elements. Alternatively the dimensionality of the phase space could increase stepwise throughout evolution, and thereby allow new dynamics to appear. In this way, we believe that vector field embryogeny not only represents an alternative for evolving a dynamic system, but also provides a new perspective on the evolution of developmental processes in general.
